# Cell-cycle-controlled radiation therapy was effective for treating a murine malignant melanoma cell line *in vitro* and *in vivo*

**DOI:** 10.1038/srep30689

**Published:** 2016-08-02

**Authors:** Keisuke Otani, Yoko Naito, Yukako Sakaguchi, Yuji Seo, Yutaka Takahashi, Junichi Kikuta, Kazuhiko Ogawa, Masaru Ishii

**Affiliations:** 1 Department of Immunology and Cell Biology, Graduate School of Medicine and Frontier Biosciences 2-2, Yamada-oka, Suita, Osaka, Japan; 2 Department of Radiation Oncology, Graduate School of Medicine, Osaka University 2-2, Yamada-oka, Suita, Osaka, Japan.

## Abstract

Radiotherapy is a commonly used regimen for treating various types of intractable cancers, although the effects depend on the cell cycle of the targeted cancer cell lines, and for irradiation purposes it is therefore critical to establish a protocol for controlling the cell cycle. Here, we showed that a common murine melanoma cell line B16BL6 was more vulnerable to irradiation during the early S phase, and that synchronisation of the cell cycle greatly increased the therapeutic effects of radiotherapy. Cell-sorting experiments, according to cell-cycle phase, using B16BL6 cells demonstrated that cells in the early S phase were the most susceptible to radiotherapy. Gemcitabine, a clinically utilised anti-cancer drug, induced cell-cycle arrest during the early S phase in B16BL6 cells, and thus a synergistic therapeutic effect was observed when irradiation was administered at the right time. Human pancreatic cancer cell line PANC-1 exhibited similar properties to B16BL6 in terms of its radiosensitivity during the S/G2/M phase and also demonstrated a synergistic effect of cell cycle synchronisation. These results show the importance of cell-cycle control in the application of irradiation and suggest a suitable time interval between chemotherapy and radiotherapy, as well as providing useful information for treating intractable cancer.

Radiotherapy is a major therapeutic approach in the treatment of cancer, together with surgery and chemotherapy. Such treatments are recommended to each patient depending on the origin and histological features of their particular type of cancer, as well as the progression of the disease, including the patient’s overall status. Radiotherapy often plays a part in cancer therapy, although it becomes problematic when cancer cells show resistance to irradiation. For example, malignant melanoma demonstrates radio-resistance, and irradiation is therefore not a good choice for treating such cancers, except when used as an adjuvant or for palliative therapy.

The main irradiation target is DNA, leading to double-strand breaks (DSBs). Repair of DSBs is performed via homologous recombination or nonhomologous end joining. Upregulation of the DNA damage response is associated with radio-resistance^1^,^2^. The outcome of irradiation is affected by the cell cycle^3^,^4^. Mitotic cells are hypersensitive to irradiation, presumably because they inactivate DSB repair^5^. Inactivating DSB repair during mitosis is assumed to inhibit telomere fusion^6^. During interphase, cell survival is maximal when cells are irradiated during the early post-mitotic (G1) and pre-mitotic (G2) phases of the cycle and is minimal during the mitotic (M) and late G1 or early DNA synthesis (S) phases^4^. Successive studies have claimed that this trend varies depending on the cell line^7^,^8^,^9^. In these studies, the cell cycle was synchronised at the M phase, and cell-cycle progression was analysed by uptake of radioisotope-labelled thymidine. Radiosensitivity during each cell-cycle phase was analysed in bulk. However, the interval between mitosis and DNA synthesis comprises the G1 and G0 phases. Therefore, the duration of the G0/G1 phase is variable, making detection of S-phase entry difficult. Radiosensitivity within the cell cycle, considering the variability of G0/G1 phase duration, has not been well investigated in the literature.

Recently, a fluorescent labelling technique known as fluorescent ubiquitination-based cell-cycle indicator (Fucci) has enabled visualisation of the cell cycle in living cells^10^. In this study, we used the Fucci system to reveal the critical association between radiosensitivity and the cell cycle. Some of the chemotherapeutic agents currently in use affect the cell-cycle distribution, and we therefore evaluated the effective timing and combination of irradiation and chemotherapy.

## Results

### Basic characterisation of a Fucci-expressing B16BL6 melanoma cell line

**Figure 1:**
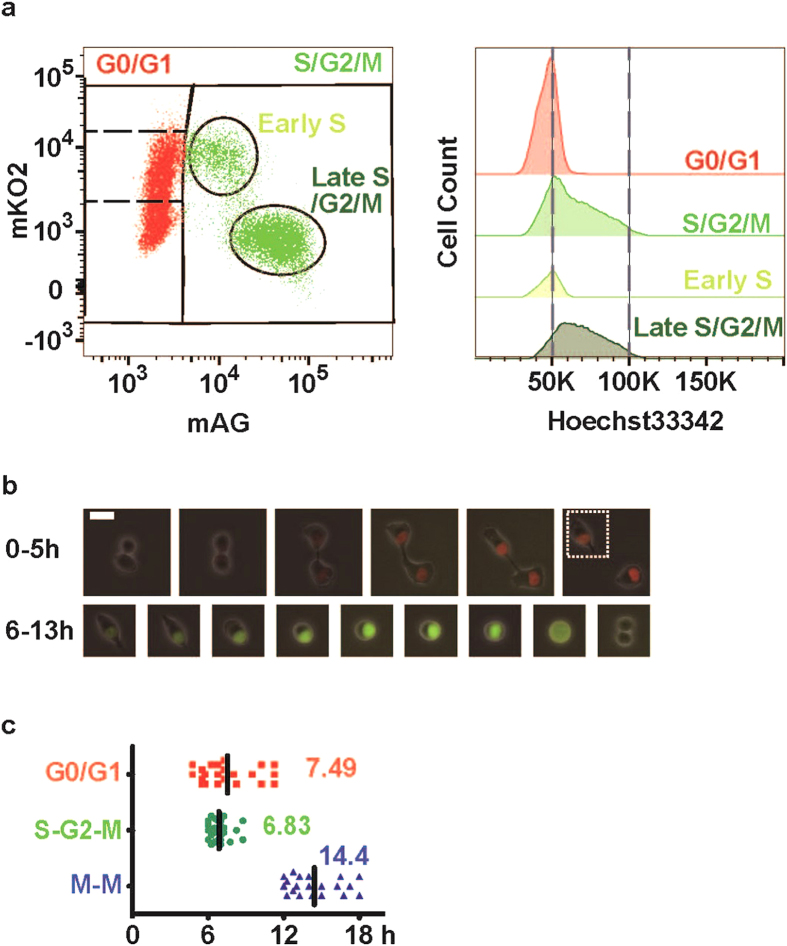
Fucci introduction into the murine melanoma cell line B16BL6. (**a**) Cell-cycle analysis of Fucci-expressing B16BL6 murine melanoma cells by flow cytometry. First, the cells were divided into three subpopulations: mAG-hGeminin-negative, mAG-hGeminin-positive, and mKO2-Cdt1-mAG-hGeminin double-positive. The DNA content of each population was analysed by staining with Hoechst 33342 dye (histograms). The coloured lines in the right-hand panels represent histograms of the cells shown in the left-hand panels. These data confirm that mAG-negative cells (i.e. G0/G1 cells) have 2N-DNA content, while mKO2-positive and mAG-positive cells (i.e. early S cells) have greater DNA content, and mKO2-negative and mAG-positive cells (i.e. late S/G2/M cells) even greater DNA content. The mAG-hGeminin-negative population was further divided into mKO2-Cdt1-negative, -moderately positive, and -highly positive cells (i.e. the early-, late-, and retained-G0/G1 phases, respectively). (**b**) Representative images of cell-cycle changes in Fucci-expressing B16BL6 cells visualised by time-lapse imaging. Post-mitotic cells displayed no fluorescent signal but gradually fluoresced red. When cells entered S phase (square), the cells fluoresced green and underwent mitosis. The time course was measured in 1-h intervals, except for the last two images (15-min intervals). A time-lapse movie can be seen in Supplementary Video 1. Scale bar: 20 μm. (**c**) Dot plots of the cell-cycle intervals analysed by time-lapse imaging. By monitoring cell colour, the interval of each cell-cycle phase was analysed. Entry into S phase was indicated by a green fluorescent signal, and the timing of mitosis was indicated by the appearance of a new cell. Values indicate the average interval.

We introduced Fucci to the B16BL6 murine melanoma cell line, which forms highly radio-resistant tumours^11^. Fucci-expressing B16BL6 cells could be divided into at least three different subpopulations, including mAG(−), mKO2(+) mAG(+), and mKO2(−) mAG(+), and further staining with Hoechst 33342 (representing the DNA contents) showed that these populations corresponded to the G0/G1, early S, and late S/G2/M phases, respectively (Fig. 1a). Because the red signal reflected the duration of the G0/G1 phase, cells in that phase could be divided into mKO2(−)mAG(−) and mKO2(+)mAG(−), corresponding to the early and late G0/G1 phases. Then we set the retained G0/G1 population within the red-emitting population on an area with a stronger red signal compared to the yellow populations. Using time-lapse imaging analyses, the cell-cycle changes in individual cells could be monitored (Fig. 1b, Supplementary Video 1), and the durations of the G0/G1 and S/G2/M phases under normal conditions were calculated to be 7.53 ± 0.46 and 6.87 ± 0.12 h, respectively (Fig). The period from one mitosis to the next mitosis (M-M) corresponded well with the sum of the durations of the G0/G1 and S/G2/M phases, demonstrating the accuracy of this measurement.

### Identification of a radiosensitive cell-cycle phase in B16BL6 melanoma cells and other cell lines

**Figure 2:**
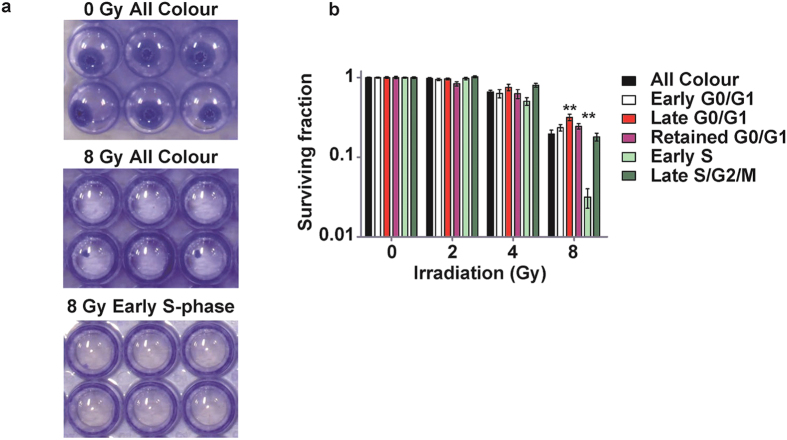
Detecting vulnerability of the cell-cycle phase to irradiation by cell sorting. For colony-formation assay with respect to the cell cycle, suspended Fucci-expressing B16BL6 cells were irradiated and sorted immediately into 96-well plates according to their cell colour. Colonies formed after 14 days were counted (**a**). All data were normalised by the 0 Gy condition. The parent-gated population is shown as the “All Colour” population. Statistical comparisons with the “All Colour” population were performed by Dunnett’s test. The early S-phase population was significantly radiosensitive at 8 Gy and the late G0/G1 phase population was significantly radioresistant (**b**). Bar: SEM, *p < 0.05.

It has been suggested that the therapeutic effect of irradiation is affected by the cell-cycle phase^3^, although this remains controversial. To compare the radiosensitivity of B16BL6 with other cell lines, we analysed the human cell lines of cervical cancer HeLa, colorectal cancer HCT 116, pancreatic cancers PANC-1 and MIA PaCa-2, and osteosarcoma MG-63. Their overall survival fraction after 5 Gy irradiation is shown in Supplementary Figure S1, which confirmed that B16BL6 was prominently radioresistant among the cell lines used. To clearly identify the radiosensitive phase, B16BL6 and the other cells were sorted according to cell cycle phase (marked by Fucci), and colony-formation assays were conducted using the respectively-collected fractions in response to irradiation (Fig. 2a,b, Supplementary Figure S2). As for the G0/G1 phase, the radiosensitivity plateaued or decreased as the cell cycle progressed towards the late G0/G1phase in all cell lines (Fig. 2b, Supplementary Figure S2). While radiosensitivity might vary depending on the cell lines at the retained G0/G1 phase, all of the cell lines except for B16BL6 had almost died after 5 Gy irradiation. However, B16BL6 remained radioresistant against 8 Gy in the late G0/G1 phase (Figs 2b and S2). Nevertheless, it became significantly sensitive to 8 Gy irradiation during the early S phase (Fig. 2b). This tendency towards early S phase vulnerability during the S/G2/M phase was marginally observed in PANC-1 and MG-63 but was not statistically significant, presumably because of the high radiosensitivity of these cell lines. These results demonstrate that radiation therapy can be most effective when the irradiation is applied during the appropriate cell cycle phase; in this case, during the early S phase.

### Effect of gemcitabine on B16BL6 *in vitro* and *in vivo*

**Figure 3:**
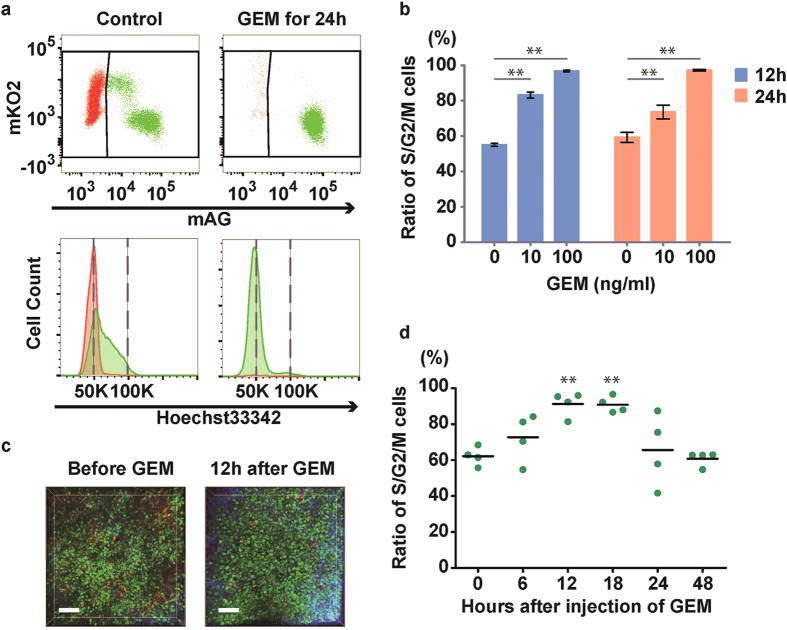
Effect of gemcitabine *in vitro* and *in vivo*. Analysis of the effect of gemcitabine (GEM) on the cell cycle *in vitro* by flow cytometry (**a**). Fucci-expressing B16BL6 cells were incubated with gemcitabine and subjected to flow cytometry. Compared with the control condition, gemcitabine increased the percentage of green-fluorescing cells, and those cells had almost 2N-DNA content, indicating they were in early S phase. (**b**) Ratio of S/G2/M cells. Columns represent means ± SEM. Data are derived from three independent experiments. (**c**) Representative images of the *in vivo* analysis of the effect of gemcitabine on the cell cycle by two-photon microscopy. Fucci-expressing B16BL6 cells were inoculated into the abdomen of C57BL/6J mice and were treated by gemcitabine. Mice were sacrificed at the indicated time point and observed by two-photon microscopy. (**d**) Compared with the control, gemcitabine gradually increased the percentage of green-fluorescing cells, which was highest 12–18 h after treatment (p < 0.05). Each plot reflects one xenograft and the bars represent the average percentage. The statistical difference was analysed by Dunnett’s test.*p < 0.05, **p < 0.01.

Gemcitabine induces cell-cycle arrest during early S phase but the actual ratio of cells in the early S phase and G0/G1 phase was not identified^12^. Incubation with gemcitabine for 12 and 24 h increased the proportion of B16BL6 cells in the S/G2/M phase (Fig. 3a upper panel, b), which was identified as early S phase by Hoechst 33342 staining (Fig. 3a, lower panel). In the presence of 100 ng/ml gemcitabine, almost all of the cells were synchronised in S phase after 12 h (Fig. 3b). The peak of the 2N-DNA content induced by gemcitabine predominantly consisted of early S phase. To evaluate the effect of gemcitabine on the cell cycle *in vivo*, Fucci-expressing B16BL6 cells were inoculated into mice and observed by two-photon microscopy (Fig. 3c). This revealed that the percentage of green-fluorescing S/G2/M cells was approximately 60% under control conditions and increased 12 h after administration of gemcitabine (Fig. 3d). This increase was maintained until 18 h, after which the cells returned to steady-state conditions by 24 h, upon the rapid exit of gemcitabine from the cell^13^. This indicated that *in vivo* cell cycle synchronisation by gemcitabine was possible but limited during the time course and that a time point of 12 h after gemcitabine treatment would be a good window for optimising the therapeutic effect of irradiation on melanoma cells *in vivo*.

### Synergistic therapeutic effect of gemcitabine and irradiation

**Figure 4:**
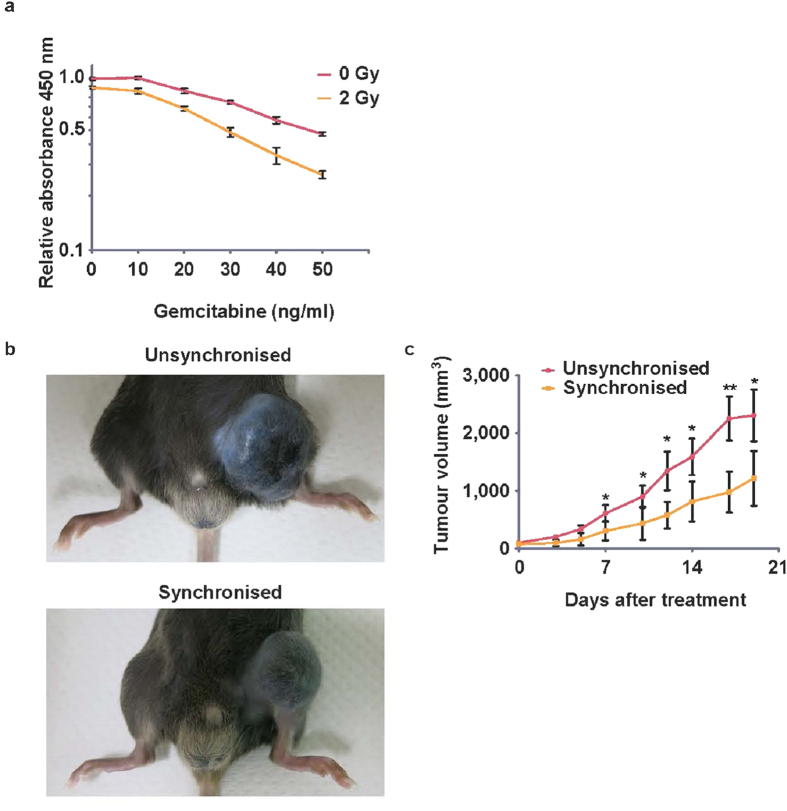
Synergetic effect of gemcitabine and irradiation *in vitro* and *in vivo*. (**a**) For *in vitro* analysis, a proliferation assay was performed. After seeding 300 cells per well of a 96-well plate for more than 2 h, the conditioned medium was changed to medium containing gemcitabine. After 12 h, the plate was irradiated and the medium replaced with gemcitabine-free medium and incubated for 5 days. All data were normalised to the 0 Gy/gemcitabine-free condition. The synergistic effect was ana**l**ysed using a generalised linear model, which revealed that not only gemcitabine and irradiation alone but also their combination significantly impaired proliferation (p < 0.01, p < 0.01, and p = 0.034, respectively). Data are derived from three independent experiments. (**b**) Representative images for *in vivo* analysis. B16BL6 cells were inoculated into the left thigh and treated with irradiation and gemcitabine. Mice treated by gemcitabine before irradiation were defined as the synchronised group, while mice irradiated before gemcitabine treatment were defined as the unsynchronised group. Xenografts grew rapidly and mice with too large xenografts were euthanised. (**c**) Compared with the unsynchronised group, the growth of xenografts in the synchronised group was prevented significantly. (n = 11 for the synchronised group, n = 10 for the unsynchronised group) bar: SEM, *p < 0.05.

To evaluate the possible synergistic therapeutic effect of gemcitabine and irradiation, we performed both *in vitro* and *in vivo* analyses, as follows. First, B16BL6 cells were incubated *in vitro* with gemcitabine for 12 h to synchronise their cell cycles during the radiation-sensitive early S phase, and they were then irradiated. Cell proliferation was measured 5 days after irradiation. The synergistic effect was assessed by a generalised linear model. Irradiation and gemcitabine individually were found to be effective at suppressing cell proliferation, and in combination, they significantly suppressed cell proliferation synergistically (p = 0.034) (Fig. 4a). The synergistic effect was also assessed by a least squares method and was also found to be statistically significant (p = 0.038). This synergistic effect was also observed in PANC-1, whose radiosensitivity within the S/G2/M phase resembled that of B16BL6 (Supplementary Figure S3a; p = 0.028 using a generalised linear model; p = 0.026 using a least-squares method). This time, we chose 24 h as the synchronisation period for *in vivo* synchronisation (Supplementary Figure S3b), which was conducted as per Fig. 3d.

Next, we evaluated the synergistic effect *in vivo*. To synchronise the cell cycle, 333 mg/kg gemcitabine was administered intraperitoneally 12–15 h before irradiation (synchronised group). For the unsynchronised group, gemcitabine was administered 8–10 h after irradiation. Treatment was repeated weekly up to three times. The increase in xenograft size was significantly suppressed in the synchronised group compared with the unsynchronised group (Fig. 4b,c). Similarly, *in vivo* treatment of the PANC-1 xenograft revealed the rapid shrinking of the tumour on day 3 (Supplementary Figure S3c). These results demonstrate that cell-cycle-controlled therapy, such as early S-phase arrest induced by gemcitabine followed by irradiation, would be very effective for some types of cancers.

## Discussion

Using the Fucci reporter system, we found that cells in early S phase are susceptible to radiotherapy in B16BL6 melanoma. The conventional technique used to synchronise the cell cycle in the M phase is affected by the varying duration of the G0 or G1 phase, which was also seen in our results (Fig. 1c). Due to this variation, despite synchronisation in M phase, cells collected in early S phase were likely to be contaminated with cells in late G0/G1 phase. In addition, other synchronisation techniques such as aphidicolin or double thymidine block induce cell damage. As the cell recovers from the damage, the cell cycle progresses in synchronisation. The Fucci system has the advantage of detecting entry of the cell into S phase by the emergence of geminin and persistent expression of Cdt1 (a DNA replication factor) without the use of toxic drugs. Using a cell sorter, the cell-cycle phase in each cell can be selected precisely according to the duration of the G0/G1 phase. In this manner, we found that B16BL6 cells showed high radiosensitivity during the early S phase and that both PANC-1 and MG-63 had a similar tendency.

We also achieved cancer cell-cycle synchronisation using gemcitabine. Gemcitabine arrests the cell cycle at early S phase^12^. Irradiation and gemcitabine are reported to have a synergistic effect^14^. The mechanism underlying this synergistic effect is reported to inhibit DNA repair, while depletion of the dCTP pool, chromosomal aberrations, and modification of intracellular metabolism have additionally been proposed as mechanisms^15^,^16^,^17^,^18^. We have provided a cell-line-specific point of view and proposed that the effect of gemcitabine on cell-cycle synchronisation is another synergistic mechanism of radiation and gemcitabine in cancers and affects a weak point during early S phase. Further research is necessary before these findings can be applied clinically. One reason is that radiosensitivity within the cell cycle varies depending on the cell line^7^,^8^,^9^, which we confirmed. Therefore, our results need to be advanced before they can contribute clinically; transducing cancer cells with the Fucci system might help in the evaluation of radiosensitivity in the cell cycle in patients, to determine individual drug responses^19^,^20^. Another method would be to compare radiosensitivity and gene profiles in various cancers, and identify the key patterns influencing radiosensitivity.

It was only by changing the treatment order that we found a significant difference in tumour volume. Chemotherapy and radiotherapy influence the immune system, and this has an impact on further treatment outcomes. The abscopal effect, which is an additional effect of irradiation on an unirradiated tumour, is thought to be mediated by the immune system^21^. Radiation has been reported to enhance the diversity of the T-cell receptor repertoire of intratumoral T cells^22^. On the other hand, some chemotherapeutic agents, including gemcitabine, elicit the tumour protective function of macrophages^23^,^24^,^25^. Taking these findings into account, *in vivo* treatment of cell-cycle-synchronised populations may be merited by the cancellation of the effect of gemcitabine on macrophages by irradiation. As for the unsynchronised group, the cascade of antigen-presenting responses initiated by irradiation could have been impaired by an influx of gemcitabine.

As far as we are aware, there has been no report regarding treatment by irradiation and gemcitabine with cell cycle synchronisation, particularly with respect to malignant melanomas. We demonstrated for the first time that the use of real-time identification of the cell cycle stage at the single cell level in cell cycle synchronisation with gemcitabine is critically important for determining the timing of irradiation. Among the cell lines we tested, B16BL6 was shown to be the most appropriate for the study of radioresistant cancer cells, while the others were severely damaged by 5 Gy irradiation (Figures S1 and S2). The heterogeneity of cancer cells against irradiation treatment depending on the clones and cell lines is a critical issue in the application of combination therapy^26^,^27^,^28^,^29^, and therefore the accumulation of data regarding effective combination protocols in various cancer cell lines is useful for the comprehensive combination of irradiation and chemotherapy. Here, we provide the novel result that gemcitabine had almost completely synchronised by the early S phase *in vitro*, and the same result was also demonstrated *in vivo*. Radiotherapy synchronised with the cell cycle was proven to be effective *in vitro* and *in vivo* toward melanoma and pancreatic cancer cell lines.

In conclusion, the Fucci system was effective for detecting the radiosensitive phase in a specific cell line. For cells exhibiting radiosensitivity in early S phase, cell-cycle synchronisation by gemcitabine was effective both *in vitro* and *in vivo*.

## Methods

### Cell lines and reagents

The murine melanoma cell line B16BL6 was obtained from Riken BRC through the National Bio-Resource Project of MEXT, Japan. The human cervical cancer cell line HeLa, pancreatic cancer cell lines PANC-1 and MIA PaCa-2, colorectal cancer cell line HCT 116, and osteosarcoma cell line MG-63 were obtained from ATCC (Manassas, VA, USA). B16BL6 cells were cultured in Roswell Park Memorial Institute (RPMI) 1640 medium supplemented with 10% foetal bovine serum (FBS), 100 U/mL penicillin, and 100 mg/mL streptomycin and maintained at 37 °C in a humidified incubator with 5% CO_2_ in air. Other cell lines were cultured in Dulbecco’s modified Eagle’s medium (DMEM) instead of RPMI. Gemcitabine was purchased from Tokyo Chemical Industry (Japan). All cells were maintained at less than 90% confluency.

### Establishment of the Fucci cell line

The mKO2-hCdt1(1–110) and mAG-hGem (30–120) Fucci probes cloned into the CSII-EF-MCS vector^30^ were transfected into HEK293T cells using packaging plasmids^31^. The supernatant was harvested to infect B16BL6 cells. Stable transformants were selected by single-cell sorting using a Sony SH800 cell sorter (Sony, Tokyo, Japan). Fucci-green (mAG) and Fucci-red (mKO2) were excited using 488-nm and 561-nm lasers, and their emission was detected using 530/30BP and 585/42BP filters, respectively. Among the clones obtained, that showing the most balanced expression of green and red fluorescence was selected, such that the double-positive fraction reflected early S phase. The Fucci colour and phase of the cell cycle were analysed by flow cytometry using Hoechst 33342 fluorescent stain, and the DNA context was confirmed to be consistent with the Fucci colours.

### Flow cytometry

Detached cells were suspended in phosphate buffered saline (PBS) with 2% FBS and were kept on ice, except during irradiation. Hoechst 33342 was excited by a 405-nm laser, and mKO2 and mAG were excited by a 488-nm laser using FACS Canto II (BD Biosciences, New Jersey, USA). Fluorescent signals were collected at 530 nm (530/28 BP) for Hoechst 33342, at 530 nm (530/28 BP) for mAG, and at 575 nm (575/26 BP) for mKO2. Cell data were analysed using the FACS Canto II or FlowJo X 10.0.7 software (Tree Star, Ashland, OR, USA).

### Time-lapse imaging

Cells were cultured on a φ35-mm glass-bottom dish, and time-lapse imaging was performed every 15 min using a BioStation IMQ (Nikon, Tokyo, Japan), which is a small incubation unit equipped with a humidified imaging chamber set at 37 °C, 5% CO_2_ in air and with a microscope. Time-lapse images were processed by Imaris x64 (ver. 7.6.5.; Bitplane AG, Zurich, Switzerland).

### Irradiation *in vitro*

The cells were irradiated with doses of 2–8 Gy using a ^137^Cs source (Gammacell 40 Exactor; Atomic Energy of Canada Ltd., Ontario, Canada) delivering 0.84 Gy/minute. Irradiation took approximately 10 min and was conveyed at room temperature. Non-irradiated cells were also kept at room temperature.

### Colony-formation assay

Suspended Fucci-induced cells were irradiated and placed onto 96-well round-bottom plates, with a single cell in each well, using the Sony Cell Sorter and were incubated for 2 weeks. Colonies were stained with crystal violet and were then counted to determine the surviving fractions. Clones were considered to represent viable cells if they contained more than 50 cells. To analyse phase-specific radiosensitivity statistically, a Dunnett’s test was used to compare each population with an all-colour group.

### *In vitro* synergistic effect using a cell proliferation assay

B16BL6 cells were seeded into 96-well plates at a density of 3 × 10^2^/well in 200 μL of a conditioned medium. After incubation for several hours, the conditioned medium in each well was replaced with a medium containing gemcitabine (0–50 ng/ml) and incubated for 12 h. The culture plates were then irradiated, and the medium was replaced with a gemcitabine-free medium and further incubated for 5 days. Cell proliferation was assessed using a Cell Counting Kit-8 incorporating WST-8 (Dojindo Molecular Technologies, Kumamoto, Japan) following the manufacturer’s instructions. Absorbance was measured at 450 nm by a plate reader (Model 680XR; Bio-Rad, Hercules, CA, USA). A similar experiment was performed on PANC-1, but the density was 1 × 10^3^/well and incubation with GEM occurred over 24 h. The synergistic effect was analysed statistically using a generalised linear model and a least-squares method.

### Cell cycle analysis *in vivo*

All animal experiments were performed with the approval of the Animal Experiments Committee of Osaka University. The methods were carried out in accordance with approved guidelines. Two days before inoculation, the abdominal skin of C57BL/6J mice (Japan CLEA) was shaved. Fucci-expressing B16BL6 cells (5 × 10^5^ cells) were injected subcutaneously into both sides of the abdomen. One week after inoculation, gemcitabine was administered intraperitoneally at 333 mg/kg (1,000 mg/m^2^ in humans), and the animal was sacrificed at a particular time. The xenografts were fixed using 4% paraformaldehyde under protection from light. Xenografts were observed using an inverted two-photon microscope (A1R-MP, Nikon), driven by a Chameleon Vision II Ti:Sapphire laser (Coherent) tuned to 940 nm, and inverted microscopes equipped with multi-immersion objectives (CFI-Plan-Fluor, 20X/N.A. 0.75, Nikon). Images were taken at a depth of 100 μm from the tumour surface by 1-μm pitch. Acquired imaging data were analysed using automated cell detection by Imaris for red- and green-fluorescing cells with a diameter greater than 10 μm. Fucci clone cells were inoculated into Nonobese diabetic/severe combined immunodeficiency (NOD/SCID) mice to analyse PANC-1.

### *In vivo* treatment

Male C57BL/6J mice (7–8 weeks old) were randomly assigned to each treatment group. Two days before inoculation, the skin of the left thigh was shaved. B16BL6 wild type cells (5 × 10^5^ cells) were injected subcutaneously into the left thigh. Xenografts were measured three times a week, and xenografts less than 5 mm in maximum length 5 days after inoculation (day -2) were excluded from the analysis. Xenografts were irradiated 7, 14, and 21 days after inoculation (days 0, 7, 14). The xenografts were irradiated using another Gammacell 40 Exactor delivering 0.70 Gy/min. Mice were fixed in a tube wrapped with 2 mm of lead with a hole to expose the left thigh. The tube containing a mouse was further shielded between two lead-layered plates each 10 mm in thickness. This shield was calculated to block 76% of the gamma ray from ^137^Cs. The tumour volume was calculated using the following formula: tumour volume (mm^3^) = 3.14 × (large diameter) × (small diameter)^2^/6. For the analysis of PANC-1, cells were inoculated into NOD-SCID mice and the interval between irradiation and gemcitabine was 24 h. The tumour size was statistically analysed using analysis of variance (ANOVA).

### Statistical analyses

Statistical analyses were performed using JMP Pro software (ver. 10.0.2; SAS Software, CA, USA). Values are presented as means ± standard error of the mean (SEM).

## Additional Information

**How to cite this article**: Otani, K. *et al*. Cell-cycle-controlled radiation therapy was effective for treating a murine malignant melanoma cell line *in vitro* and *in vivo. Sci. Rep.* **6**, 30689; doi: 10.1038/srep30689 (2016).

## Supplementary Material

Supplementary Information

Supplementary Video 1
