# Supplementary material for: Cell-cycle-controlled radiation therapy was effective for treating a murine malignant melanoma cell line in vitro and in vivo
Source: Sci Rep. 2016 Aug 2;6:30689. doi: 10.1038/srep30689 (PMC4969753; doi:10.1038/srep30689)
Supplement: Supplementary Information [file srep30689-s1.pdf]

Cell-cycle-controlled radiation therapy was effective for treating a murine malignant melanoma cell line in vitro and in vivo

Keisuke Otani[1,2] , Yoko Naito[1], Yukako Sakaguchi[1], Yuji Seo[2], Yutaka Takahashi[2], Junichi Kikuta[1], Kazuhiko Ogawa[2], Masaru Ishii[1*]

[1]Department of Immunology and Cell Biology, Graduate School of Medicine and Frontier Biosciences, and [2]Department of Radiation Oncology, Graduate School of Medicine, Osaka University

*mishii@icb.med.osaka-u.ac.jp

Tel: +81 6-6879-3880, -3881

Fax: +81 6-6879-3889

2-2 Yamada-oka, Suita, Osaka, 565-0871, Japan

Supplementary Figure S1

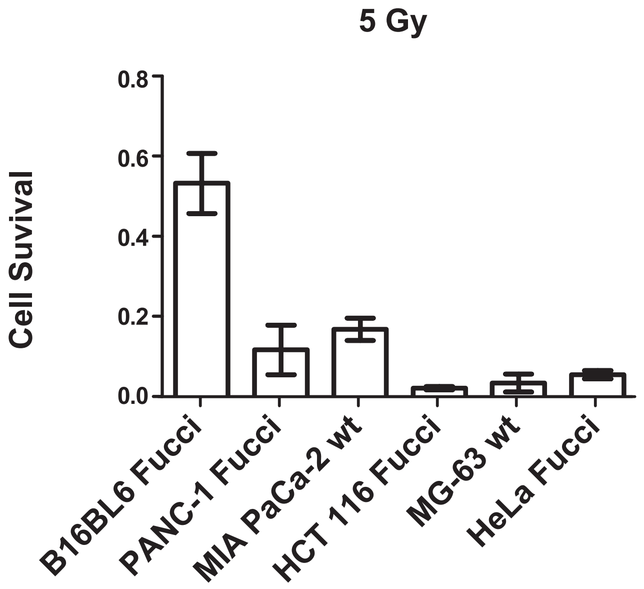

Supplementary Figure S1. Radiosensitivity in six cell lines at 5 Gy. Among the 6 cell lines evaluated, B16BL6 showed prominent radioresistance. Because cell survival after irradiation with 5 Gy could not be evaluated for the Fucci clones of MIA PaCa-2 and MG-63, survival of the wild-type cell line is shown instead. Each bar: SEM. Data are derived from three independent experiments.

Supplementary Figure S2

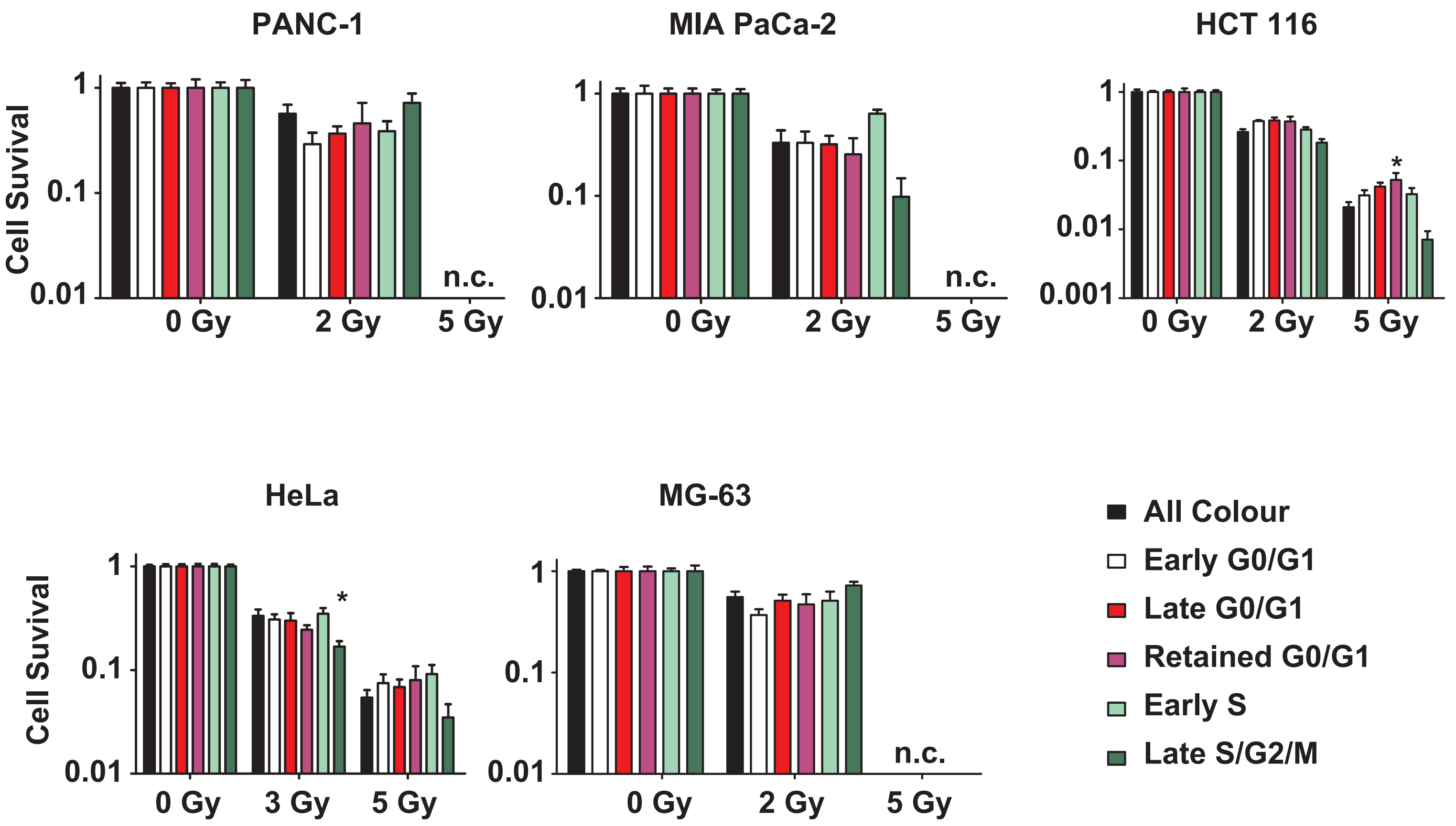

Supplementary Figure S2. Detecting the vulnerability of the cell cycle phase to irradiation by cell sorting in several cell lines.

No cell lines exhibited increased radiosensitivity during the late G0/G1 phase. With regard to the S/G2/M phase, the radiosensitive phase was variable within these cell lines. Bar: SEM, *: $p < 0.05$. Data are derived from three independent experiments.

Abbreviation: n.c.: no colonies formed.

Supplementary Figure S3

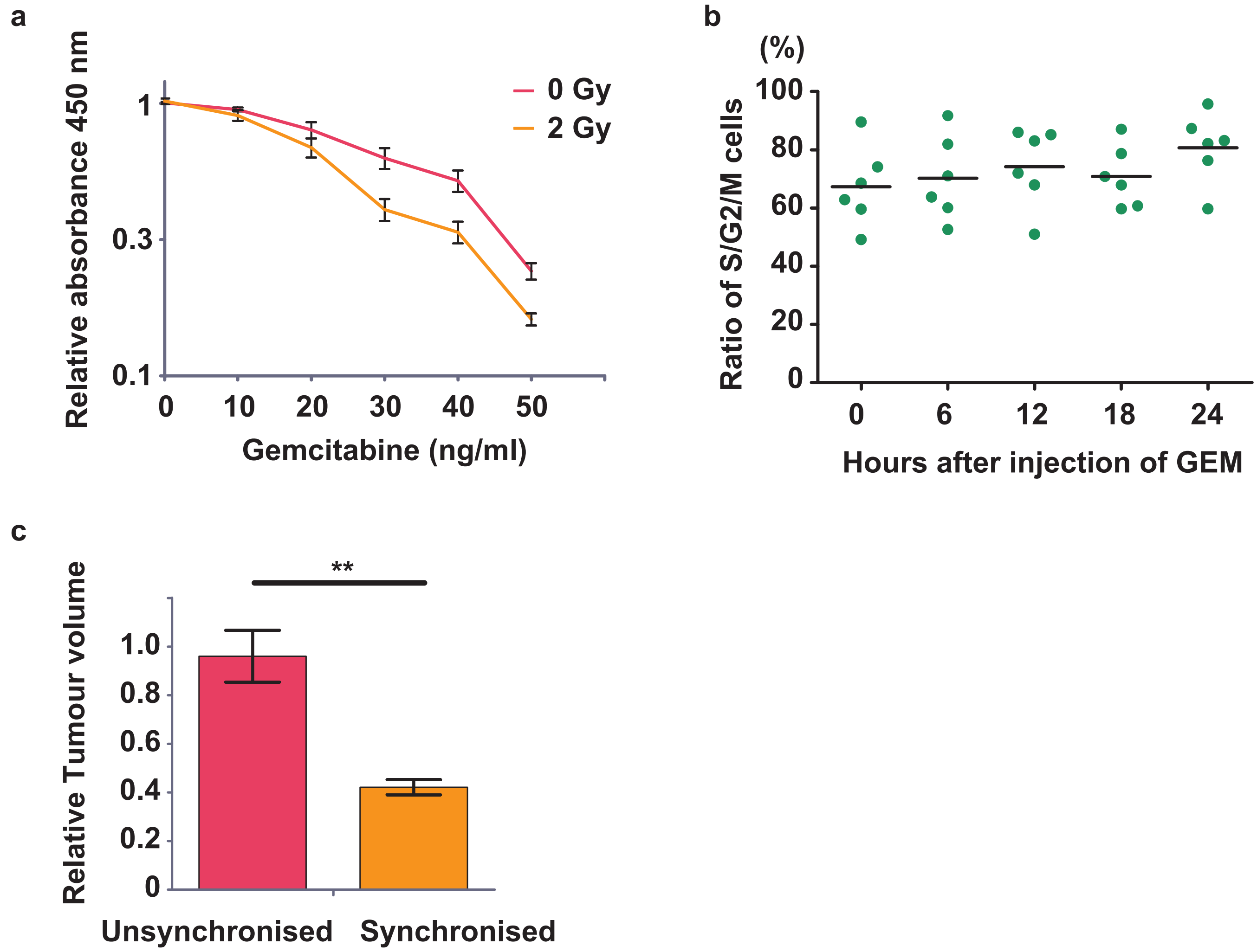

Supplementary Figure S3. Synergetic effect of gemcitabine and irradiation toward PANC-1 in vitro and in vivo.

(a) For the in vitro analysis, a proliferation assay was performed, as per Figure 4a. At this time, 1 × 103 cells per well of a 96-well plate were seeded and exposed to gemcitabine for 24 h before irradiation and then incubated for 4 days. Gemcitabine alone and a combination of gemcitabine and irradiation significantly impaired proliferation ($p < 0.01$ and $p = 0.028$ using a generalised linear model, $p < 0.01$ and $p = 0.026$ using a least-squares method, respectively). Data are derived from four independent experiments. (b) To test the intravital synchronisation of the cell cycle by gemcitabine toward PANC-1, an experiment similar to that shown in Figure 3d was performed. This time, the percentage of green-fluorescing cells was highest at 24 h after treatment. Each plot reflects one xenograft and the bars represent the average percentage. Cell cycle synchronisation in the S/G2/M phase in vivo was not statistically increased, probably due to the variety of control conditions. (c) For the in vivo analysis, xenografts were treated with irradiation and gemcitabine, as per Figure 4c. The interval between gemcitabine and irradiation was again set at 24 h. The size of the xenografts was significantly reduced 3 days after treatment in the synchronised group, compared to the unsynchronised group ($n = 7$ for the synchronised group, $n = 8$ for the unsynchronised group).

Bar: SEM, **: $p < 0.01$.

Supplementary Video 1. Time-lapse imaging of cell-cycle changes in Fucci-expressing B16BL6 cells. Representative images are shown in Fig. 1b.
